# Supplementary material for: Inappropriate Heart Rate Response to Hypotension in Critically Ill COVID-19-Associated Acute Kidney Injury
Source: J Clin Med. 2021 Mar 23;10(6):1317. doi: 10.3390/jcm10061317 (PMC8005163; doi:10.3390/jcm10061317)
Supplement: Supplementary file 1 [file jcm-10-01317-s001.pdf]

Table S1. Sensitivity analysis: linear regression using a stepwise backward selection fitting the relationship between changes in heart rate during CVVHDF-induced hypotension in critically ill patients and associated dependent variables.

Variables	Estimate * heart rate change †, bpm	95% CI	p value
COVID-19 status	−12.12	−19.52– 4.72	0.003
Change in MABP †	−0.27	−0.57–0.03	0.07
PEEP ‡	1.42	−0.38–3.22	0.11

Definitions of abbreviations: CI, confidence interval; COVID-19, Coronavirus infectious disease 2019; CVVHDF, continuous venovenous hemodiafiltration; MABP, mean arterial blood pressure; PEEP, positive end-expiratory pressure.

* Estimates the independent effect of each included variable (COVID-19 status, MABP change and PEEP level) on heart rate changes during DIH.

† Parameters at the time of CVVHDF-induced hypotension onset minus parameter at the time of last hemodynamic monitoring before CVVHDF-induced hypotension (maximum of two hours before)

‡ At the time of last hemodynamic monitoring before CVVHDF-induced hypotension (maximum of two hours before)
